# Transcriptomic and Metabolomic Profiling Reveals the Protective Effect of *Acanthopanax senticosus* (Rupr. & Maxim.) Harms Combined With *Gastrodia elata* Blume on Cerebral Ischemia-Reperfusion Injury

**DOI:** 10.3389/fphar.2021.619076

**Published:** 2021-04-16

**Authors:** Bingfeng Lin, Renhao Chen, Qi Wang, Zhifeng Li, ShiLin Yang, YuLin Feng

**Affiliations:** ^1^ Jiangxi University of Traditional Chinese Medicine, Nanchang, China; ^2^ State Key Laboratory of Innovative Drug and Efficient Energy-Saving Pharmaceutical Equipment, Nanchang, China; ^3^ Nanchang Key Laboratory of Active Ingredients of Traditional Chinese Medicine and Natural Medicine, Nanchang, China

**Keywords:** cerebral ischemia-reperfusion injury, *Acanthopanax senticosus* harms, *Gastrodia elata* blume, transcriptomic, metabonomic

## Abstract

The effects of current treatment strategies used in ischemic stroke are weakened by cerebral ischemia-reperfusion (CIR) injury. Suitable treatment regimens targeting CIR injury are still lacking. Two herbs, namely, *Acanthopanax senticosus* (Rupr. & Maxim.) Harms (ASE) and *Gastrodia elata* Blume (GEB), have been used as traditional Chinese medicine and are indicated in the treatment of stroke and cerebrovascular diseases. However, there are no studies that report the effects of ASE combined with GEB in the treatment of CIR injury. In this study, we used the Zea Longa method to induce CIR injury in male Wistar rats. Results of the pharmacodynamic studies revealed that co-administration of ASE and GEB may improve neuronal injury and prevent neuronal apoptosis by reducing oxidative stress and inflammation, and also help prevent CIR injury. On the basis of our hypothesis, we combined the results from transcriptomic and metabonomic analyses and found that ASE and GEB could prevent CIR injury by targeting phenylalanine, pyrimidine, methionine, and sphingolipid metabolism. Therefore, our study provides the basis for the compatibility and efficacy of ASE and GEB.

## Introduction

Stroke is a common clinical neurological condition that seriously endangers human health and life. It is the fifth most common cause of death in America (Mozaffarian et al., 2016). The incidence of stroke is high; approximately 7,95,000 individuals, 87% of whom are ischemic, suffer from stroke each year in the United States alone (Lloyd-Jones et al., 2009). Ischemic stroke, the most common variant, affects the nervous system at the morphological and molecular levels. Brain tissue is sensitive to ischemia and hypoxia, and early recovery of blood reperfusion is the primary focus in the clinical management of ischemic strokes (Soares et al., 2009). However, when blood is perfused into the ischemic brain tissue, inflammatory reactions and neuronal death may be intensified owing to the generation of free radicals, calcium overload, and other factors (Bai and Lyden, 2015). Alleviation of the pathophysiological process of cerebral ischemia-reperfusion (CIR) injury has become a research hotspot worldwide; however, the progress is not satisfactory. The treatment windows of the thrombolytic drugs and neuroprotective agents that are currently used for therapy are narrow and pose challenges during the therapy of stroke (Green, 2008; Boese et al., 2020).

Traditional Chinese medicine (TCM) has a long history in the treatment of stroke. Based on the pathogenesis theory of TCM and the so-called “Syndrome Differentiation and Pattern Diagnosis,” most patients who have suffered an ischemic stroke can be grouped as being deficient in Qi and wind movement (Jhong et al., 2013). Therefore, herbs with Qi-replenishing, wind-dispelling, and blood-activating effects are frequently used to reverse the damage caused by ischemic stroke (Zhang and Zhao, 2014; Lu et al., 2018). *Acanthopanax senticosus* (Rupr. & Maxim.) Harms [ASE, *Eleutherococcus senticosus* (Rupr. & Maxim.) Maxim.] is widely used as a potent Qi supplement to strengthen the spleen as a means to treat cardiovascular diseases (Xie et al., 2015). *Gastrodia elata* Blume (GEB) is a typical herbal medicine in TCM, which suppresses the hyperactive liver and reduces endogenous wind (Huang et al., 2019). ASE and GEB are herbal medicines that have been widely used for the treatment of ischemic stroke. Using data mining and the Delphi expert questionnaire, Rongrong Zhou was found that ASE-GEB may be a pair of drugs suitable for the treatment of stroke (Zhou et al., 2020). Previous studies have revealed the primary active constituents of ASE to be eleutheroside E (Bai et al., 2011), isofraxidin (Bai et al., 2011), hyperoside (Liu et al., 2012), eleutheroside B (Tohda et al., 2008), and quercetin (Pei et al., 2016), which can inhibit ischemic brain injury and protect against neuritic atrophy and cell death. The main active constituents of GEB include 4-hydroxybenzyl alcohol, gastrodin, and parishin. 4-Hydroxybenzyl alcohol ameliorates ischemic injury by inhibiting the activation of caspase-3 and increasing the expression of Bcl-2 (Yu et al., 2010). Gastrodin ameliorates oxidative stress and inflammation by activating the Akt/Nrf2 pathway (Peng et al., 2015). In addition, parishin is mainly degraded to gastrodin *in vivo* (Tang et al., 2014). ASE and GEB have been abundantly reported in the treatment of CIR injury and nerve protection; however, the combined effect and mechanism of the two drugs are not clear. In this study, we explored the efficacy of ASE combined with GEB for the treatment of CIR injury and attempted to explore the mechanism of action underlying this combination. However, as the multi-component and multi-targeting characteristics of TCM play an important role in the efficacy of this combination, the potential mechanism of the combination of ASE and GEB is not clear.

In recent decades, emerging “omics” technologies, including transcriptomics and metabonomics, have immensely improved the ability to study the biochemical changes caused by TCM interventions in biological systems. Metabolomics techniques provide insight into the mechanisms of various physiological conditions and abnormal processes at the systems level of metabolites (Johnson et al., 2016). Transcriptomics studies can be used to perform rapid qualitative and quantitative analysis of mRNA transcripts of up-stream genes (Wang et al., 2009). Therefore, the integration of transcriptomics and metabonomics is of great significance for comprehensive research to determine the efficacies of and mechanisms of the formulations used as TCM (Du et al., 2018; Wu et al., 2019).

In this study, through the combination of transcriptomic analysis and metabonomics, we revealed the mechanism of the combination of GEB-ASE in alleviating CIR injury in rats.

## Experimental Approaches

### Extraction Methods

Medicinal materials were collected from Heilongjiang Province and identified as the dried roots and rhizomes or stems of *Acanthopanax senticosus* (Rupr. & Maxim.) Harms (ASE, *Eleutherococcus senticosus* (Rupr. & Maxim.) Maxim.), and *Gastrodia elata* Blume (GEB) by Professor Guoyue Zhong of the Jiangxi University of Traditional Chinese Medicine. Voucher specimens (accession number JZ-2017GYZ-CWJ-A2 and JZ-2017GYZ-TM-A3) were deposited in the Center of National Medicine Resource, Jiangxi University of Traditional Chinese Medicine, China.

The plant materials were washed, dried, sliced, and ground to a powder. To prepare the AEGE extract, 500 g of ASE and 500 g of GEB were mixed with 5,000 ml of 70% ethanol, refluxed and extracted for 2 h. The residue was subjected to re-extraction three times using 5,000 ml of ethanol for each subsequent extraction. All fractions were mixed, filtered, concentrated at 60 C using a rotary evaporator and freeze-dried. Lastly, AEGE extract at a concentration of 200 mg/kg was prepared by adding an appropriate volume of 0.5% carboxymethyl cellulose sodium (CMCNa; Xilong Science and Technology Co., Ltd, Shantou, China) solution to the dried extract.

Ethanol extracts of AEGE were analyzed using a Shimadzu UHPLC (ESI) system (Shimadzu, Kyoto, Japan) and an AB SCIEX quadrupole time-of-flight mass spectrometer (TripleTOF® 5,600, AB SCIEX, Framingham, MA, USA). The extract was dissolved in methanol and then filtered through a 0.22-μm membrane filter. Samples were analyzed using an ACQUITY UPLC C18 column (100 mm × 2.1 mm, 1.7 µm, Waters) at 40°C. The mobile phase comprised water containing 0.1% formic acid (A) and acetonitrile (B), and the proportion of mobile phase B was as follows: 2–10%, 0–8 min; 10–20%, 8–16 min; 20–30%, 16–24 min; 30–90%, 24–29 min; 90%, 29–34 min. The injection volume was 1 µl and the flow rate was 0.3 ml/min. TOF-MS and TOF-MS/MS were performed synchronously. Mass spectra were acquired in the negative ion mode. The parameters were set as follows: turbo spray temperature, 550 C; ion spray voltage floating, -4,500 V; collision gas, -35 eV. The mass range was set at 50–1,250 Da. The obtained data were analyzed using Peak View Software (AB SCIEX, Framingham, MA, United Satets).

### Animals and Grouping

Adult male Wistar rats (240 ± 20 g) were obtained from Beijing Charles River Co. Ltd (Beijing, China; certification number: SCXK (Jing): 2016-0006). Rats were acclimated for 5 days and provided access to water and standard laboratory animal diet *ad libitum*. They were randomly divided into four groups as follows: sham, IR, AEGE, and nimodipine (Nim), with 15 rats/group. The AEGE and Nim groups received AEGE (200 mg/kg/d) or Nim (15 mg/kg/d), respectively, orally for 15 days. The sham and IR groups received 0.9% sodium chloride orally for 15 days.

All experimental procedures were performed in accordance with the ethical principles for laboratory animals of the State Key Laboratory (Reference number: BCTG-2016-18). Experiments were reviewed and approved by the Animal Care Committee of the Jiangxi University of Chinese Medicine.

### Establishment of IR Model

Rats were anesthetized using an intraperitoneal 10% injection of chloral hydrate (350 mg/kg). Cerebral ischemia-reperfusion injury was established following the method by Longa et al. (1989). Briefly, the external carotid artery was ligated and the common carotid artery was embolized through the internal carotid artery to the middle cerebral artery using a thread coated with silicone at the head end. Two hours after the induction of ischemia, the thread was pulled out to ensure the establishment of reperfusion. Rats in the sham group underwent a similar surgery, but without insertion of the monofilament.

### Pharmacodynamics of AEGE

#### Neurobehavioral Assessment and Evaluation of Cerebral Infarction

After 24 h of reperfusion, the behavioral score of rats was evaluated using a 0–4 point scoring system as follows: no observable neurological dysfunction was marked as 0 points; toe curled up powerless was marked as 1 point; inclined to crawl in the opposite direction was marked as 2 points; turned autonomously to the opposite side upon being stimulated with slight sound was marked as 3 points; fell to the opposite side and had no spontaneous activity for a long time was marked as 4 points.

After neurobehavioral evaluation, the rats were anesthetized using chloral hydrate. Blood was collected from the posterior abdominal aorta. The animals were euthanized by decapitation and the brain tissues were collected.

Brain tissues of 3 rats/group were randomly selected for triphenyl tetrazolium chloride (TTC) staining. The entire brain tissue was sectioned into five 2 mm-thick coronal slices. These sections were incubated at 37 C for 16 min in 1% TTC solution (Solarbio, Beijing, China) and fixed with 4% paraformaldehyde for 3–5 h. The total brain slices and infarcted area were stained and imaged using Image-Pro Plus 6.0 (Media Cybernetics, Bethesda, MD, United states) and the percentage of the infarcted area was calculated.

#### H&E Staining

The brain tissues of 2 rats from each group were randomly selected for H&E staining. The portion between the root of the crossed optic nerve and the quadrigeminal body of the brain was placed in 4% paraformaldehyde at 4 C for about 24 h. After the fixed brain slices were dehydrated and embedded to prepare continuous paraffin sections, HE (Solarbio, Beijing, China) staining was performed and the samples were observed using fluorescence microscopy (Leica, Weztlar, Germany).

#### Biochemical Evaluation

The brain tissues of 7 rats from each group were randomly selected for biochemical evaluation. The blood on the surface of the brain was washed with saline, and the excess saline was blotted using filter paper. Next, the coronal sections around the bregma point were collected (1 mm). Coronal brain tissues were weighed after sectioning. Brain tissue was added to precooled saline in a 1:9 ratio, homogenized, and centrifuged for 10 min (4,000 rpm). The activities of SOD, GSH-Px, and MDA in brain tissue were measured using the respective assay kits (Jiancheng Bioengineering, Nanjing, China). The IL-10, IL-1β, and TNF-α levels in brain tissue were determined using ELISA (Neobioscience Technology, Shenzhen, China).

### Metabolomics Analysis

#### Plasma Pretreatment and UPLC-Q/TOF-MS Analysis

The plasma samples of 10 rats in the sham, IR, and AEGE group were randomly selected for metabonomics analysis. Blood was collected from the abdominal aorta and loaded into an Eppendorf tube containing heparin sodium. The supernatant was obtained by centrifugation at 4,000 rpm for 10 min. Plasma supernatants were treated with methanol containing 2-chloro-L-phenylalanine (10 μg/ml) in a 4:1 ratio (methanol: plasma supernatant, v/v, 250 μl). After centrifugation at 4 C (4,000 rpm, 10 min), the supernatant was collected for analysis. A 50-μl aliquot of each centrifuged supernatant was uniformly mixed to yield a quality control (QC) sample.

A Shimadzu UHPLC (ESI) system and an AB Sciex quadrupole time-of-flight mass spectrometer (TripleTOF 5600) were used for LC-MS analysis. An ACQUITY UPLC C18 column (100 mm × 2.1 mm, 1.7 µm, Waters) was used for all analyses. The mobile phase was a mixture of 0.1% formic acid in water (A) and acetonitrile (B). The proportion of mobile phase B was as follows: 2–30%, 0–3 min; 30–60%, 3–5 min; 60–80%, 5–15 min; 80–100%, 15–16 min; 100%, 16–19 min; 100–2%, 19–20 min; 2%, 20–25 min. The injection volume was 2 μl, the flow rate 0.3 ml/min, and the column temperature was 40 C. TOF-MS and TOF-MS/MS were performed synchronously. Mass spectra were acquired in the positive and negative ion modes. The parameters of the positive ion mode were set as follows: ion spray voltage floating, 5,500 V; collision gas, 40 eV. The parameters of the negative ion mode were set as follows: ion spray voltage floating, −4,500 V; collision gas, −40 eV. The turbo spray temperature was 550 C and the TOF-MS mass ranged from m/z 50–1,250 Da.

During analysis of the sample sequence, a QC sample was run after every five injections to validate the analytical methodology (Zhang et al., 2017). QC (n = 6) and plasma samples were analyzed using LC-MS. Data quality was evaluated based on the relative SDs (RSDs) of the retention times and intensities of 10 typical peak (including internal standard) of the QC samples.

#### Data Analysis

Raw data of plasma samples were analyzed using MarkerView 2.0 (AB SCIEX, Framingham, MA, United States). Before chemometric analysis, the data obtained for each sample were normalized using internal standards (2-chloro-L-phenylalanine). The features were subjected to statistical analysis only when detection frequencies of any group reached 100% and the RSD was less than 30%. The missing values were replaced by half minimum of abundances of features. The pre-processed data were analyzed using principal component analysis (PCA) and orthogonal partial least squares discriminant analysis (OPLS-DA) using SIMCA 14.1 (Umetrics, Umeå, Sweden). Additionally, the model was considered to qualify when the cumulative values of R2 (score of raw data interpreted by the model) and Q2 (predictive power of the model) were greater than or equal to 0.5; OPLS-DA was performed using a permutation test (200 permutations) to avoid overfitting.

Features with variable importance in projection (VIP) scores >1 in the OPLS-DA model and *p*-values < 0.05 in *t*-test were selected and their corresponding metabolites identified. Human Metabolome database (HMDB) was used as a tool for the identification of differential metabolites using LC-MS. Associated metabolic pathways were established using the Kyoto Encyclopedia of Genes and Genomes (KEGG) and MetaboAnalyst 3.0, in addition to other online tools.

### Transcriptomics Studies

#### Library Preparation for Transcriptome Sequencing

The brain tissues of 3 rats from the sham, IR, and AEGE groups were randomly selected for transcriptome analysis. Total RNA was extracted from about 150 mg of brain tissue using a MirVana total RNA extraction kit (Ambion, Carlsbad, CA, United Sates). RNA concentration was measured using Qubit® RNA Assay Kit and a Qubit® 2.0 Fluorometer (Life Technologies, Carlsbad, CA, United States). RNA integrity was assessed using an RNA Nano 6,000 Assay Kit of the Bioanalyzer 2,100 system (Agilent Technologies, CA, United States). A total amount of 1.5 µg of RNA per sample was used as the follow-up test material for RNA-sample preparation. Whole transcriptome profiling was performed using NEBNext® Ultra™ RNA Library Prep Kit for Illumina® (NEB, Ipswich, MA, United States) according to the manufacturer’s protocol.

#### Clustering, Sequencing, and Quantification of Gene Expression

Clustering of index-coded samples was performed using a CBOT Cluster Generation system using HiSeq 4,000 PE Cluster Kit (Illumina). Next, RNA sequencing (150 bp, pair-ends) was performed using standard Illumina HiSeq 4000 platform protocols. All downstream analyses were based on clean data with high quality. Gene FPKM was calculated by adding the FPKM of each genome transcript. The differential expression of the two conditions was analyzed using DESeq2R software package (1.26.0). For genes with FPKM values ≥1 in at least one sample, the significantly different expressions between groups were determined according to the criteria as follows: *p* < 0.05 and |log2FoldChange| ≥ 0.58.

#### GO and KEGG Enrichment Analysis of Differentially Expressed Genes

Functional Annotation Bioinformatics Microarray Analysis (DAVID) database (Huang et al., 2009) was used to obtain Gene Ontology (GO) enrichment analysis of DEGs. GO terms (*p* < 0.05) were considered significantly enriched by DEGs. The enrichment of DEGs in KEGG pathways was tested using KEGG Orthology Based Annotation System v3.0 software; *p*-values were calculated using one-way ANOVA based on the normalized dataset. KEGG terms (*p* < 0.05) were considered significantly enriched by DEGs.

### Western Blotting

Western blotting was used to determine the protein expression of the key genes measured using transcriptomic analysis. About 100 mg of brain tissue was lyzed with 1 ml of RIPA buffer (Beyotime, Shanghai, China). The protein concentration of the brain homogenate was measured using a bicinchoninic acid assay kit (Thermo Scientific, MA, United States). Protein samples were resolved using sodium dodecyl sulfate polyacrylamide-gel electrophoresis and transferred onto polyvinylidene difluoride membranes (Millipore, Schwalbach, Germany). Then, the proteins were immunoblotted with their corresponding primary and secondary antibodies. A chemiluminescence assay kit was used to determine the intensity of each band using a Tanon 6600 luminous imaging workstation (Tanon, Shanghai, China). Protein expression was determined using Image-Pro Plus 6.0.

### Statistical Analysis

All experimental data were analyzed using Student’s *t*-test or one-way ANOVA test with SPSS version 21.0 software (IBM, Armonk, NY, United States) package to calculate statistical significance. *p* < 0.05 was considered statistically significant.

## Results

### Identification of Chemical Constituents in AEGE

The total negative ion chromatograms of AEGE are shown in Supplementary Figure S1. A total of 18 components were characterized (Supplemental Table S1) and their chemical structures are shown in Supplementary Figure S2. Four major active components, isofraxidin, eleutheroside E, gastrodin and parishin A, were identified by comparison with the standards, whereas the others were identified by checking their characteristic product ions (Supplementary Figure S1 **)**.

### Pharmacodynamic Effects of AEGE

#### Neurobehavioral Scores and Evaluation of Cerebral Infarction

**FIGURE 1:**
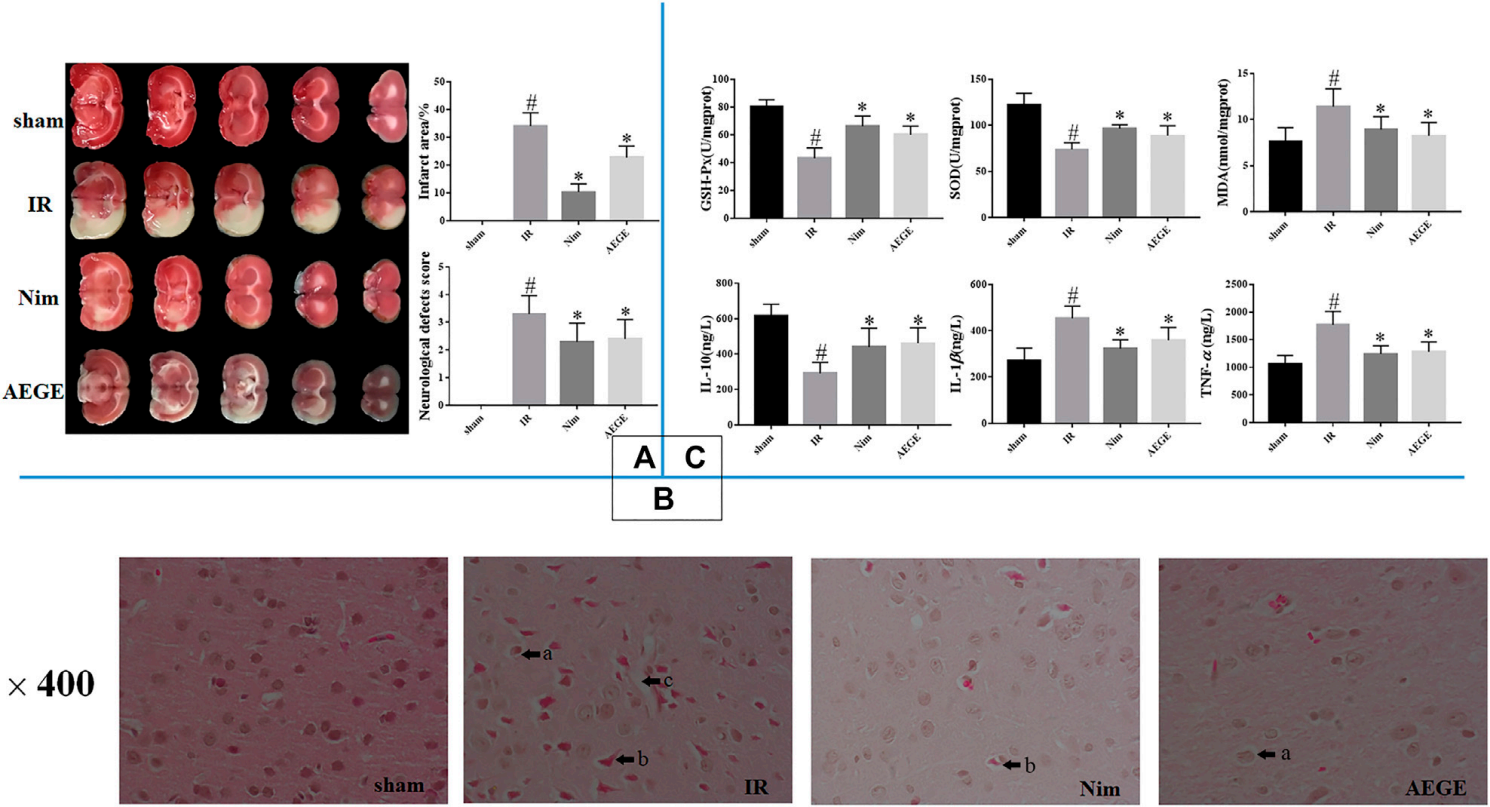
Changes in brain histopathology and biochemical indices in rats with cerebral ischemia-reperfusion injury. **(A)** TTC staining, neurological defects, and infarct area. **(B)** H&E staining. The area marked with an “a” indicates peripheral demyelination; “b” indicates nuclear pyknosis, nerve cell atrophy, and neuronal degeneration; and “c” indicates cytoclasis of the brains in MCAO/R rats. **(C)** Changes in SOD, GSH-Px, MDA, IL-10, IL-1β*,* and TNF-α levels in rat brains. Data are expressed as mean ± SD. “#” represents sham group *vs*. the IR group (#*p* < 0.05); “*” represents IR group *vs*. the AEGE group (*p < 0.05).

The neurobehavioral scores of the rats in the IR group were significantly higher (*p* < 0.05) than those in the sham group. The cerebral infarction area was consistent with these scores. TTC staining (Figure 1A) and scoring scales indicated the successful establishment of the IR model. The rats in the AEGE group showed significantly lower scores (*p* < 0.05) and infarction areas (*p* < 0.05) compared to those in the IR group. These findings indicated that AEGE and Nim had a therapeutic effect on nerve injury and CIR injury.

#### HE Staining

HE staining (Figure 1B) showed no neuronal damage or inflammatory cell infiltration in the cerebral cortex of rats in the sham group. However, the ischemic cortex of the brains exhibited cytoclasis, nuclear pyknosis, nerve-cell atrophy, neuronal degeneration, and peripheral demyelination in the IR group. Treatment with AEGE and Nim improved the histopathological features caused due to CIR injury. The neuronal cells were restored to normal, and there were only a few nerve cells that exhibited peripheral demyelination and nerve-cell atrophy.

#### Biochemical Evaluation

SOD and GSH-Px activity in the brain tissue of IR rats decreased significantly (*p* < 0.05), whereas there was a significant increase in MDA levels (*p* < 0.05) compared to the sham group. Compared to that in the IR group, GSH-Px and SOD activity increased significantly (*p* < 0.05) and MDA level decreased significantly (*p* < 0.05) in the AEGE group (Figure 1C).

In rats with IR injury, IL-10 expression was found to decrease significantly (*p* < 0.05), and the levels of IL-1β and TNF-α were significantly elevated (*p* < 0.05) compared to the corresponding values in the sham group. A significant decrease in TNF-α and IL-1β levels (*p* < 0.05) and a significant increase in IL-10 level (*p* < 0.05) was observed in the AEGE group (Figure 1C) compared to that in the IR group. These findings suggested that AEGE may be efficacious in treating cerebral ischemia-reperfusion injury by reducing oxidative stress and inflammation.

### Metabolomics Studies

**FIGURE 2:**
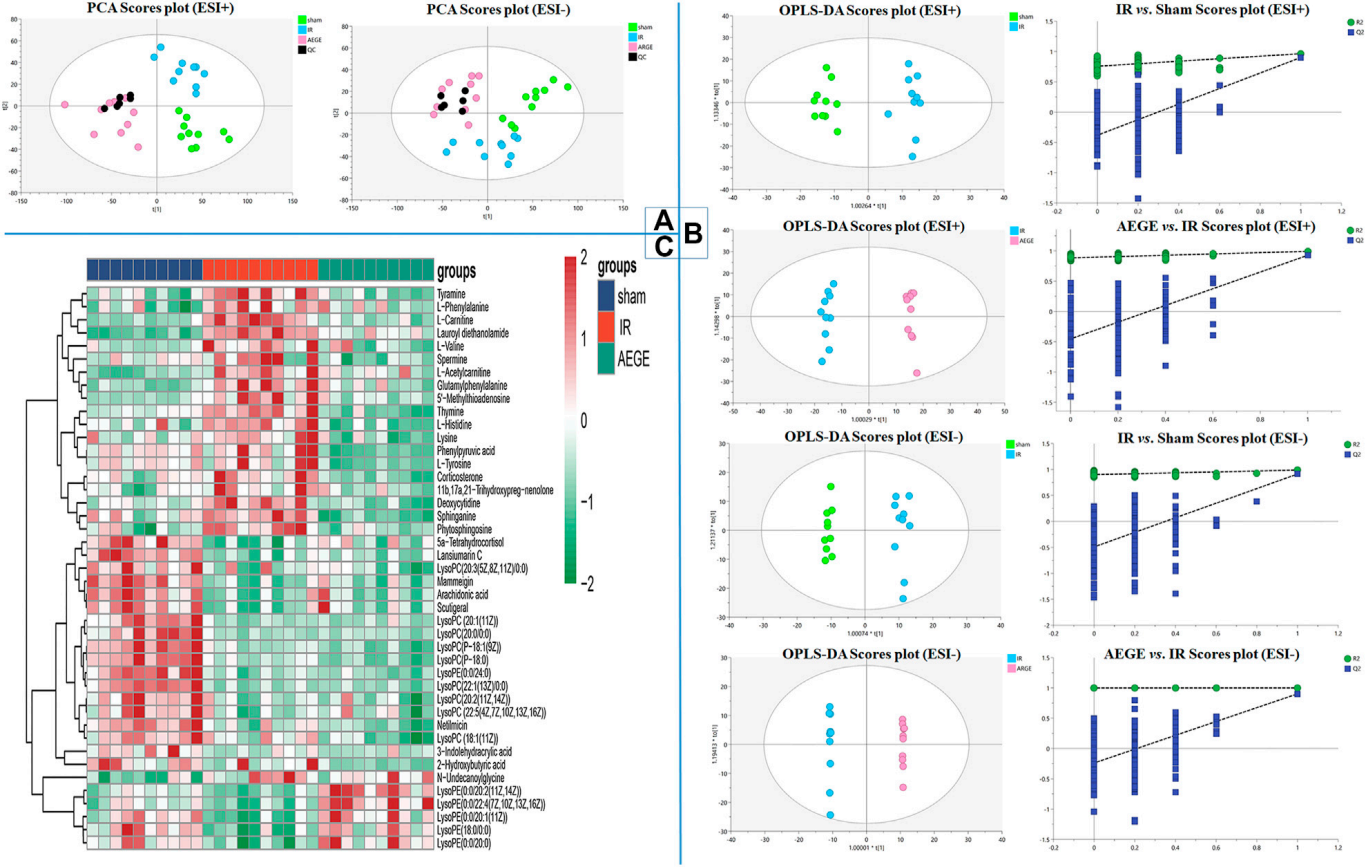
Signatures of plasma metabolism in rats. **(A)** PCA score plot for the validation of quality control (QC). **(B)** OPLS-DA and permutation score plots of sham, IR, and AEGE groups. **(C)** Heatmap of metabolites in the cerebral ischemic injury of rats in the sham, IR, and AEGE groups. Red for higher level and blue for lower level.

The 10 typical ion peaks in the QC samples showed low RSD in the peak intensity and retention times in the positive and negative ion modes (Supplementary Figure S3; Supplemental Table S3). The clustering of QC samples and significant separation between the sham, IR, and AEGE groups in PCA (Figure 2A) confirmed the robustness and reproducibility of the test method. Moreover, OPLS-DA modes both exhibited well-verifiable parameters (Supplemental Table S4). The permutation test with 200 iterations showed that the OPLS-DA models were not overfitted (Figure 2B).

The metabolites (VIP > 1.0, *p* < 0.05) were considered as potential differential metabolites. Combined with the primary parent ion information and secondary ion fragment information collected using high-resolution mass spectrometry, differential metabolites were identified by matching Peakview1.2 with the substance molecules in HMDB, and combined using the standard sample atlas information.

Using this protocol, 43 differential metabolites (Table 1) were identified among the control, model, and AEGE groups. To better capture the changes in metabolism, a heatmap was constructed to represent the graphical view using all groups (Figure 2C). The changes in the levels of observed metabolites were divided into four scenarios as follows: 1) After administration of AEGE, the levels of metabolites, including L-phenylalanine, tyramine, deoxycytidine, sphinganine, 5′-methylthioadenosine, corticosterone, thymine, L-histidine, and phytosphingosine, returned to the same level as that in the sham group. 2**)** The metabolite levels in the IR vs. sham groups were not significantly upregulated or decreased; however, the levels decreased significantly after administration of AEGE. The types of metabolites in this category are more complex and there may be some positive feedback regulation that is considered normal. Examples include phenylpyruvic acid, L-tyrosine, and spermine. 3) The metabolite levels in the IR *vs*. sham groups were significantly upregulated or decreased; however, these levels were not significantly upregulated or decreased after the administration of AEGE. Examples include 3-indolehydracrylic acid, N-undecanoylglycine, and netilmicin. 4) The metabolite levels in the IR group were significantly downregulated compared to those in the sham group; however, after the administration AEGE, these levels were still significantly downregulated. This phenomenon was called abnormal regulation. The metabolites mainly comprise lipids and fatty acids, such as LysoPC [20:1 (11Z)/0:0], LysoPC (P-18:0), and LysoPC (P-16:0/0:0).

**TABLE 1:** Potential biomarkers of cerebral ischemic injury post-treatment with AEGE.

M/Z	Rt	Formula	Name	Adduct	HMDB	IR *vs*. sham	AEGE *vs*. IR
100.0756	2.02	C_5_H_11_NO_2_	l-Valine	+H-H_2_0	HMDB0000883	↑	↓
120.0806	2.01	C_8_H_11_NO	Tyramine	+H-H_2_0	HMDB0000306	↑***	↓***
127.0498	1.74	C_5_H_6_N_2_O_2_	Thymine	+H	HMDB0000262	↑***	↓***
146.059	1.92	C_9_H_7_NO	Lysine	+H	HMDB0000182	↑*	↓***
147.0443	1.22	C_9_H_8_O_3_	Phenylpyruvic acid	+H-H_2_0	HMDB0000205	↑	↓***
162.1109	0.92	C_7_H_15_NO_3_	l-carnitine	+H	HMDB0000062	↑***	↓****
178.0595	0.84	C_6_H_9_N_3_O_2_	l-Histidine	+Na	HMDB0000177	↑***	↓***
182.08	1.2	C_9_H_11_NO_3_	l-Tyrosine	+H	HMDB0000158	↑	↓***
188.0698	6.75	C_11_H_11_NO_3_	3-Indolehydracrylic acid	+H-H_2_0	HMDB0059765	↓*	↓
203.2234	0.81	C_10_H_26_N_4_	Spermine	+H	HMDB0001256	↑	↓***
204.1232	1.22	C_9_H17NO_4_	l-acetylcarnitine	+H	HMDB0000201	↑***	↓*
226.1788	15.89	C_13_H_25_NO_3_	N-Undecanoylglycine	+H-H_2_0	HMDB0013286	↑	↓
250.0795	1.21	C_9_H_13_N_3_O_4_	Deoxycytidine	+Na	HMDB0000014	↑***	↓***
288.2544	9.05	C_16_H_33_NO_3_	Lauroyl diethanolamide	+H	HMDB0032358	↑***	↓***
295.1275	3.3	C_14_H_18_N_2_O_5_	Glutamylphenylalanine	+H	HMDB0029156	↑***	↓***
298.0962	2.72	C_11_H_15_N_5_O_3_S	5′-methylthioadenosine	+H	HMDB0001173	↑***	↓***
302.3049	8.96	C_18_H_39_NO_2_	Sphinganine	+H	HMDB0000269	↑	↓***
318.3026	8.98	C_18_H_39_NO_3_	Phytosphingosine	+H	HMDB0004610	↑***	↓***
329.2121	7.06	C_21_H_30_O_4_	Corticosterone	+H-H_2_0	HMDB0001547	↑*	↓***
347.2222	7.05	C_21_H_32_O_5_	11b,17a,21-trihydroxypreg-nenolone	+H-H_2_0	HMDB0006760	↑*	↓*
476.3109	9.42	C_21_H_41_N_5_O_7_	Netilmicin	+H	HMDB0015090	***↑	↓
506.3621	10.18	C_26_H_52_NO_6_P	LysoPC [P-18:1 (9z)]	+H	HMDB0010408	***↑	↓
508.3734	11.45	C_26_H_54_NO_6_P	LysoPC(P-18:0)	+H	HMDB0013122	***↑	↓*
522.3512	10.58	C_26_H_52_NO_7_P	LysoPC [18:1 (11Z)]	+H	HMDB0010385	↑	↓
546.3526	15.24	C_28_H_52_NO_7_P	LysoPC [20:3 (5Z,8Z,11Z)/0:0]	+H	HMDB0010393	↓*	↓
548.3694	11.46	C_28_H_54_NO_7_P	LysoPC [20:2 (11Z,14Z)]	+H	HMDB0010392	↓	↓
550.3856	13.13	C_28_H_56_NO_7_P	LysoPC [20:1 (11Z)]	+H	HMDB0010391	↓***	↑
552.3995	15.09	C_28_H_58_NO_7_P	LysoPC(20:0/0:0)	+H	HMDB0010390	↓***	↑
566.4153	16.61	C_29_H_60_NO_7_P	LysoPE (0:0/24:0)	+H	HMDB0011497	↓***	↓
570.3534	11.46	C_30_H_52_NO_7_P	LysoPC [22:5 (4Z,7Z,10Z,13Z,16Z)]	+H	HMDB0010402	↓	↑
578.4174	15.68	C_30_H_60_NO_7_P	LysoPC [22:1 (13Z)/0:0]	+H	HMDB0010399	↓***	↓
103.0406	1.59	C_4_H_8_O_3_	2-Hydroxybutyric acid	-H	HMDB0000008	↓	↓*
164.0702	2.00	C_9_H_13_NO_3_	l-phenylalanine	-H_2_O-H	HMDB0000068	↑	↓*
303.2339	14.95	C_20_H_32_O_2_	Arachidonic acid	-H	HMDB0001043	↓***	↑
347.221	15.26	C_21_H_34_O_5_	5a-Tetrahydrocortisol	+H-H_2_0	HMDB0000526	↓***	↑
353.1403	14.55	C_21_H_22_O_5_	Lansiumarin C	-H	HMDB0034838	↓***	↑
371.2212	14.95	C_23_H_32_O_4_	Scutigeral	-H	HMDB0030012	↓***	↓
403.1574	14.94	C_25_H_24_O_5_	Mammeigin	-H	HMDB0030785	↓***	↑
480.3112	10.16	C_23_H_48_NO_7_P	LysoPE (18:0/0:0)	-H	HMDB0011130	↓***	↑
504.3122	9.84	C_25_H_48_NO_7_P	LysoPE [0:0/20:2 (11Z,14Z)]	-H	HMDB0011483	↓	↑***
506.3291	10.95	C_25_H_50_NO_7_P	LysoPE [0:0/20:1 (11z)]	-H	HMDB0011482	↓	↑***
508.3408	12.78	C_25_H_52_NO_7_P	LysoPE (0:0/20:0)	-H	HMDB0011481	↓	↑***
528.3108	9.61	C_27_H_48_NO_7_P	LysoPE [0:0/22:4 (7Z,10Z,13Z,16Z)]	-H	HMDB0011493	↓	↑***

*p < 0.05.

**p < 0.01.

***p < 0.001.

**FIGURE 3:**
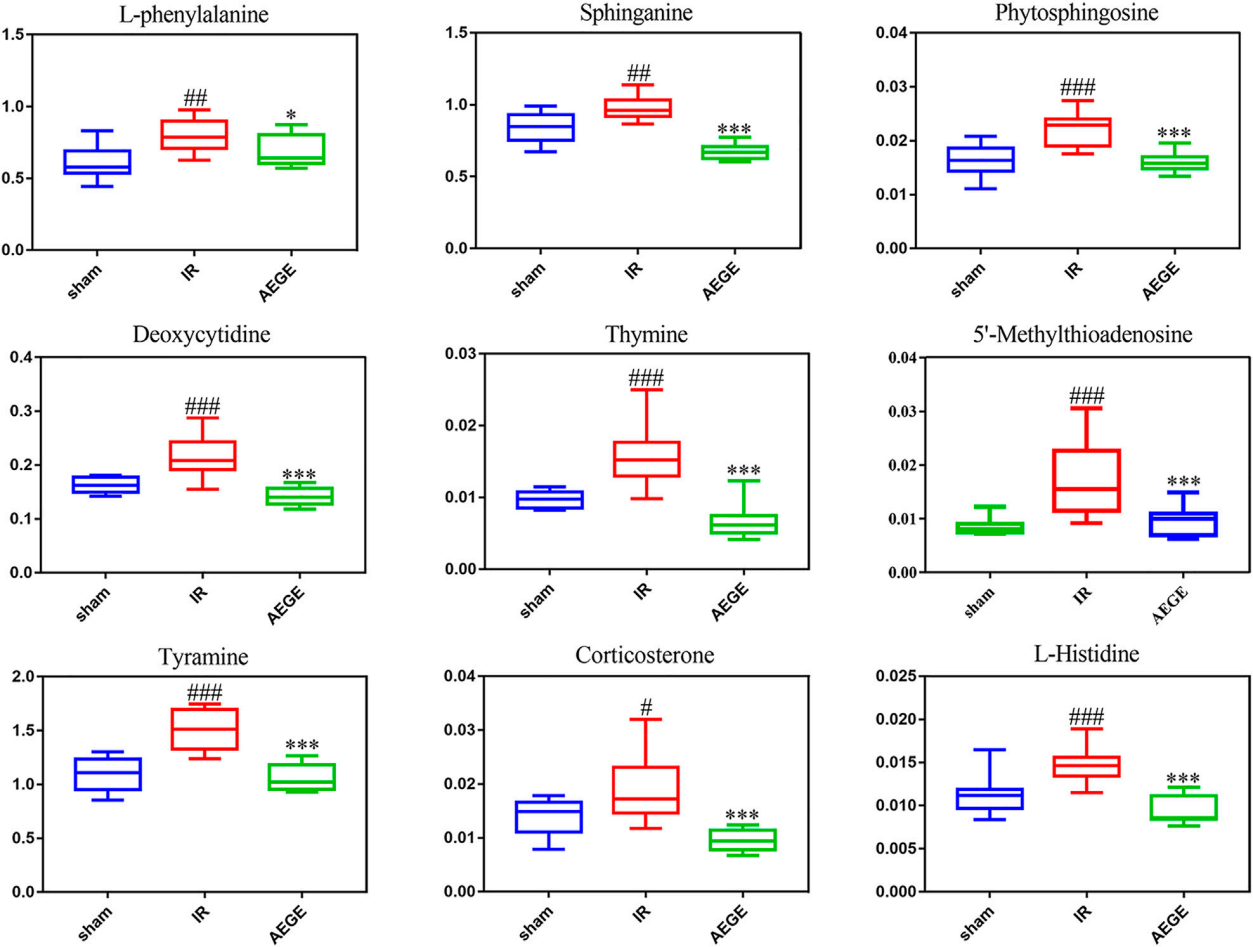
Box charts of the relative trends of metabolites involved in the important metabolic pathways that were identified. Data are expressed as the mean ± SD. “#” represents sham group *vs*. the IR group (#*p* < 0.05, ##*p* < 0.01, ###*p* < 0.001); “*” represents IR group *vs*. the AEGE group (*p < 0.05, ***p < 0.001).

Forty-three differential metabolites were enriched using the MetaboAnalyst 4.0 database to obtain the enrichment map of the metabolic pathways. The main pathways were identified for the following: phenylalanine, tyrosine, and tryptophan biosynthesis; phenylalanine metabolism; histidine metabolism; sphingolipid metabolism; pyrimidine metabolism; cysteine and methionine metabolism; tyrosine metabolism; steroid-hormone biosynthesis (Supplementary Figure S4). The levels of nine potential biomarkers, including L-phenylalanine, tyramine, deoxycytidine, sphinganine, 5′-methylthioadenosine, corticosterone, thymine, l-histidine, and phytosphingosine were significantly different in the plasma of rats in the IR group (Figure 3).

### Transcriptomics Studies

**FIGURE 4:**
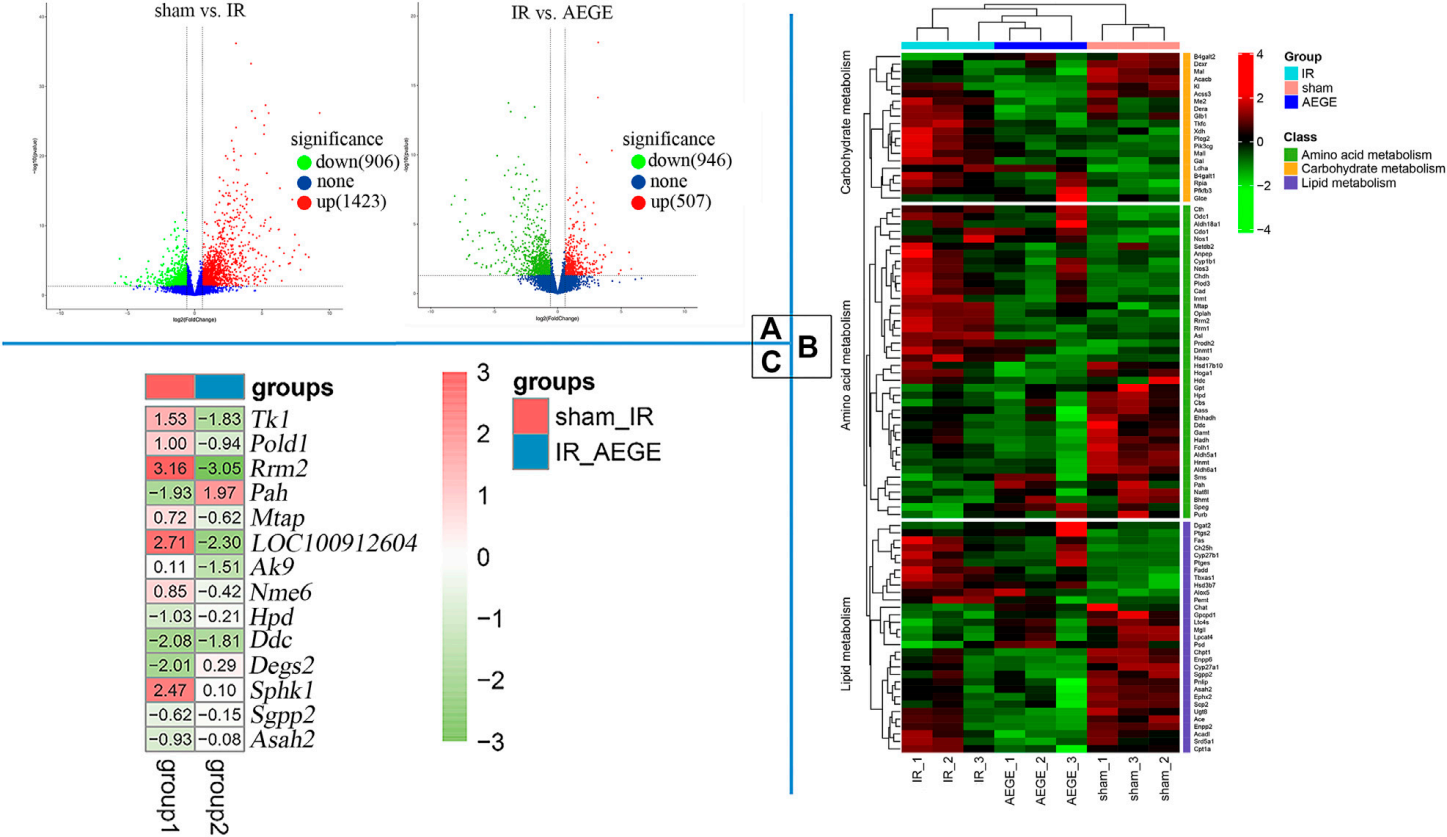
Transcriptomics for CIR injury induced by middle cerebral artery occlusion. **(A)** Volcano plots drawn for all identified genes. **(B)** Cluster analysis of DEGs and heatmap according to DEGs type of pathways. Red for higher level and green for lower level. **(C)** Heatmap of restored genes after treatment with AEGE.

A total of 2,326 DEGs were identified, which included 1,423 upregulated and 903 downregulated genes when the IR and sham groups were compared. A total of 1,453 DEGs were identified, of which 507 were upregulated and 946 were downregulated when the AEGE and IR groups were compared; among them, the expression levels of 494 genes were restored after treatment with AEGE. All genes were used to construct volcano maps (Figure 4A). It was found that many genes were upregulated in the IR group, whereas, after treatment with AEGE, the expression of most genes was significantly downregulated. The general trend in the number of DEGs is shown in Figure 4B and was classified using the KEGG database. Following AEGE administration, the expression levels of amino acid-, carbohydrate-, and lipid-related genes partially returned to a level similar to that of the sham group.

The cellular processes and functions of the significantly altered genes can be understood by using the GO database. At the level of biological processes, genes were involved in the positive regulation of apoptosis. At the level of cellular components, the significantly changed genes mainly existed in the cytoplasm. At the molecular-function level, the genes were involved in the positive regulation of ATP binding, protein homodimerization, and identical protein binding (Supplementary Figure S5).

The KEGG database was used to analyze the biological pathways mediated by the identified genes in more depth. A total of 116 pathways were found to be significantly enriched when the sham and IR groups were compared (Supplemental Table S5), whereas 28 pathways were found to be significantly enriched when the AEGE and IR groups were compared (Supplemental Table S6). Combined with metabonomic analysis, we found that the pathways of the DEGs were well associated with those of the metabolites produced during phenylalanine, pyrimidine, methionine, and sphingolipid metabolism. When the control, model, and AEGE groups were compared, the expression of six genes, including Tk1, Pold1, Rrm2, Pah, Mtap, and LOC100912604, was found to be restored (Figure 4C). Compared to the sham group, the expression levels of Hpd, Ddc, Degs2, Sgpp2, Asah2, Nme6 decreased significantly, whereas that of Sphk1 was significantly upregulated in the IR group. Following AEGE treatment, the expression of *AK9* was significantly upregulated compared to that in the IR group.

### Validation of Changes in Protein Expression

**FIGURE 5:**
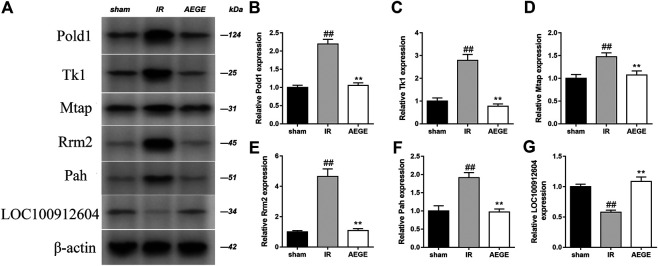
Determination of protein levels in brain tissue of rats in the sham, CIR, and AEGE groups. Expression of Pold1 **(A,B)**, Tk1, **(A, C)**, Mtap **(A,D)**, Rrm2 **(A,E)**, Pah **(A,F)**, and LOC100912604 **(A,G)** on the indicated group were detected using western blotting. Results are expressed as mean ± SD for three individual experiments which, were performed in triplicate for each condition. “#” represents sham group vs. the IR group (##*p* < 0.01); represents IR group vs. the AEGE group (**p < 0.01).

Integrated analysis indicated that the treatment of CIR with AEGE is related to phenylalanine, pyrimidine, methionine, and sphingolipid metabolism. The protein expression of the key genes in rat brains was evaluated using western blotting (Figure 5). Compared to the sham group, the expression level of Pold1, Tk1, Mtap, Rrm2, and Pah increased significantly, whereas that of LOC100912604 decreased significantly in the brain tissue of rats in the IR group. After the administration of AEGE, the protein levels returned to a level similar to that in the sham group. The protein expression of the key genes analyzed using western blot was highly consistent with the results obtained using transcriptomics analysis.

## Discussion

Several studies have reported that oxidative stress and inflammation lead to apoptosis, autophagy, and necrosis after CIR. The decreased activities of SOD and GSH-Px and the increased levels of MDA in the brain tissue of IR rats may lead to weakened activity of antioxidant enzymes in the rat brain, which in turn can disrupt the balance between the antioxidant enzyme system and free oxygen radicals (Zhan and Yang, 2006). The activities of inflammatory cytokines, IL-1β and TNF-α, in the brain tissue of IR rats increases significantly, whereas that of IL-10 decreases significantly. This results in the induction of neutrophil adhesion, migration, and activation, and leads to an increase in inflammation and destruction of the brain tissue (Zhang et al., 1998). Therefore, it is believed that rats administered AEGE as a prophylactic might be less susceptible to CIR injury. This may be owing to the antioxidant and anti-inflammatory mechanisms of AEGE.

**FIGURE 6:**
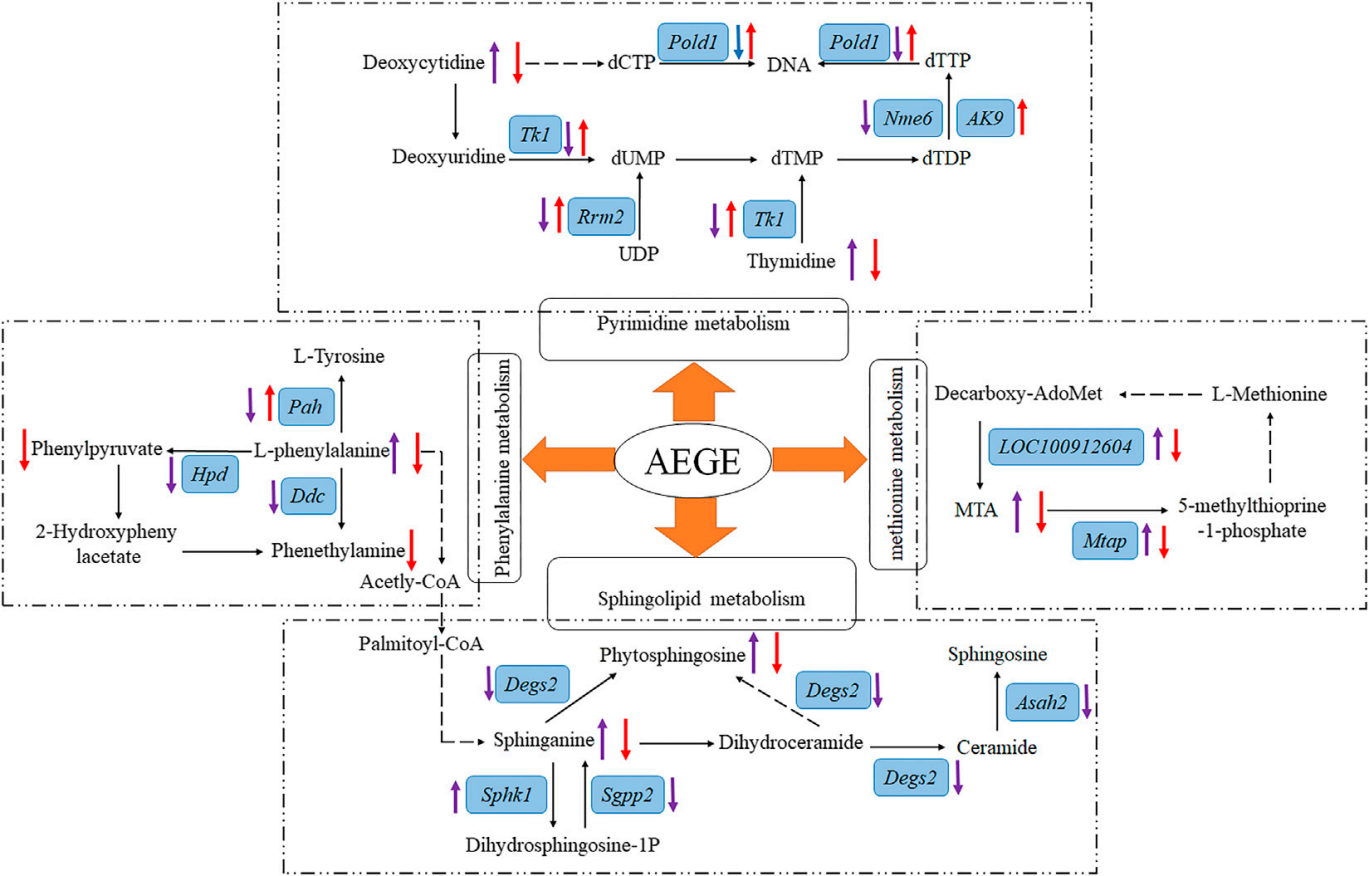
Overview of metabolic pathway analysis. After treatment with AEGE, the concentration or expression of metabolites and genes (blue background and italic) were changed (“↑” means upregulated; “↓” means downregulated; purple arrow represents the ratio of IR to sham groups; red arrow represents the ratio of AEGE to IR groups). Dashed arrow indicates multiple steps in the pathway.

To further establish the relationship between the results from transcriptomic and metabolomics studies, data of the potential metabolites and the altered genes were integrated. The main metabolic pathways of the following metabolites were identified: phenylalanine, pyrimidine, methionine, and sphingolipids. AEGE could treat CIR injury by affecting these pathways at both transcriptional and metabolic levels. Details of the metabolic pathways are shown in Figure 6.

L-phenylalanine is an essential aromatic amino acid that plays a key role in the biosynthesis of other amino acids (Pardridge, 1998). When present in sufficiently high levels, phenylalanine acts as a neurotoxin that disrupts or attacks neural cells and tissues. Plasma l-phenylalanine levels can be increased in rats with traumatic brain injury (Vuille-Dit-Bille et al., 2012). Phenylpyruvic acid is a keto-acid, which is an intermediate or catabolic byproduct of phenylalanine metabolism. Tyrosine is an amino acid synthesized from phenylalanine in the presence of phenylalanine hydroxylase. We found the metabolite levels of L-phenylalanine to be significantly higher and the expression levels of Pah, Ddc, and Hpd genes to be significantly lower than those in the IR group. However, the levels of the metabolites, phenylpyruvic acid, and L-tyrosine, were not significantly different compared to those in the sham group. This may have occurred because of the decrease in L-phenylalanine metabolism due to CIR, which led to the accumulation of L-phenylalanine, causing subsequent damage to the brain cells. After administration of AEGE, the levels of L-phenylalanine, phenylpyruvic acid, and L-tyrosine decreased significantly, and the expression of the Pah gene increased significantly. The expression trend of Pah protein analyzed using western blot was consistent with the results obtained using transcriptomics. Therefore, it could be inferred that AEGE can decrease L-phenylalanine levels. Reduced levels of L-phenylalanine may reduce the oxygen consumption of the brain, energy production, and storage, all of which are beneficial in brain recovery through phenylalanine metabolism.

Phytosphingosine exerted strong cytotoxic effects, modulated the *Caenorhabditis elegans* muscarinic acetylcholine receptor-mediated signal transduction pathway, and induced cell death (Lee et al., 2001). Sphinganine is a bioactive compound involved in cell proliferation, differentiation, transcription, autophagy, and apoptosis, all of which are relevant to inflammatory disease (Merrill et al., 2009). In this study, we found that sphinganine and phytosphingosine accumulated significantly in rats in the IR group, causing damage to brain cells. Moreover, we found that the expression of Degs2, Sgpp2, and Asah2 was significantly decreased, and that of the *Sphk1* gene was increased significantly in the brain tissue of rats with IR. This could likely be a protective mechanism of the body to defend itself against high concentrations of sphinganine and phytosphingosine, to convert sphinganine to the less toxic dihydrosphingosine-1P, and reduce the conversion of sphinganine to phytosphingosine. After treatment with AEGE, the levels of phytosphingosine and sphinganine decreased significantly compared to that in the IR group. This reduction may be attributed to the regulation of phenylalanine metabolism by AEGE, which led to the decrease in acetyl-CoA levels. Therefore, the therapeutic effects of AEGE may be achieved by regulating the levels of sphingomyelin and sphingosine in sphingolipid metabolism by regulating phenylalanine metabolism.

Deoxycytidine is one of the principal nucleosides of DNA and is composed of cytosine and deoxyribose. Thymine is one of the pyrimidine bases of living matter. Exogenous uridine and cytidine play a role in maintaining brain function in rats with CIR injury (Löffler et al., 2018). In the IR group, the levels of deoxycytidine and thymine increased significantly, and the expression of Tk1, Pold1, Rrm2, and Nme6 genes decreased significantly compared to that in the sham group. The consistent trends of the two metabolites might be closely related to the enzymes controlled by the genes integrated with DNA. Following AEGE treatment, the expression of Tk1, Pold1, Rrm2, and AK9 were found to be significantly upregulated. Therefore, it could be reasonably deduced that AEGE regulates the balance between dCTP and dTTP through genes including Tk1, Pold1, Rrm2, AK9, and Tyms. Moreover, the expression trend of Tk1, POLD1, and Rrm2 proteins, which were verified using western blotting was the same as that obtained using metabonomics analysis. This result explains the decrease of blood deoxycytidine and thymine levels after treatment with AEGE. Collectively, these findings suggested that AEGE could improve the disorder in pyrimidine metabolism caused by CIR injury.

5′-Methylthioadenosine (MTA) can yield 5-methylthioprine-1-phosphate and adenine through the metabolism of S-methyl-5′-thioadenosine phosphorylase (Mtap), which is an important step in the methionine- and purine-recovery pathways. MTA affects the regulation of gene expression and plays a role in proliferation, differentiation, and apoptosis (Ansorena et al., 2002). Protein methionine oxidation potentiates the activation of NF-κB and contributes to CIR injury (Gu et al., 2016). In this study, we found that LOC100912604, Mtap, and MTA were significantly upregulated, which may have accelerated the metabolism and oxidation of methionine after CIR. After treatment with AEGE, LOC100912604, Mtap, and MTA were significantly downregulated, which restored methionine metabolism and reduced the damage to brain cells resulting from methionine oxidation. The expression trend of Mtap analyzed using western blotting was consistent with the results of transcriptomics analysis. Thus, AEGE intervention may regulate methionine metabolism and likely contribute to its therapeutic effects.

The main limitation of this study concerning the metabolomics component is one which is common to all metabolomic investigations - identification of metabolites. Because of the unavailability to obtain the reference compounds, identifying them remained an enormous difficulty.

## Conclusion

In summary, rats pretreated with AEGE suffered less severe CIR injury. Our findings revealed that AEGE significantly reduced the area of cerebral infarction caused by CIR injury. The mechanism of AEGE in alleviating CIR was via the reduction of oxidative stress and inflammation.

Results of transcriptomic and metabonomic studies indicated that six metabolites, namely, sphinganine, thymine, phytosphingosine, l-phenylalanine, deoxycytidine, 5′-methylthioadenosine; and six genes, namely, Tk1, Pold1, Rrm2, Pah, Mtap, and LOC100912604*,* were significantly altered in the IR group. The representative proteins in the altered pathways were determined using Western blot. These metabolites and genes could be regulated after treatment with AEGE, suggesting that the therapeutic effect of AEGE in CIR may be related to the regulation of phenylalanine and pyrimidine metabolic pathways, sphingolipid metabolism, and methionine metabolism.

Although this new prescription of ASE combined with GEB is seldom used in a clinical setting, it provides useful clues for the further development of this herbal combination.

## Data Availability

The datasets generated for this study can be found in the Sequence Read Archive, https://www.ncbi.nlm.nih.gov/gds/?term=GSE160500.
