# Supplementary material for: Transcriptomic and Metabolomic Profiling Reveals the Protective Effect of Acanthopanax senticosus (Rupr. & Maxim.) Harms Combined With Gastrodia elata Blume on Cerebral Ischemia-Reperfusion Injury
Source: Front Pharmacol. 2021 Apr 16;12:619076. doi: 10.3389/fphar.2021.619076 (PMC8085551; doi:10.3389/fphar.2021.619076)
Supplement: Supplementary file 1 [file datasheet1.docx]

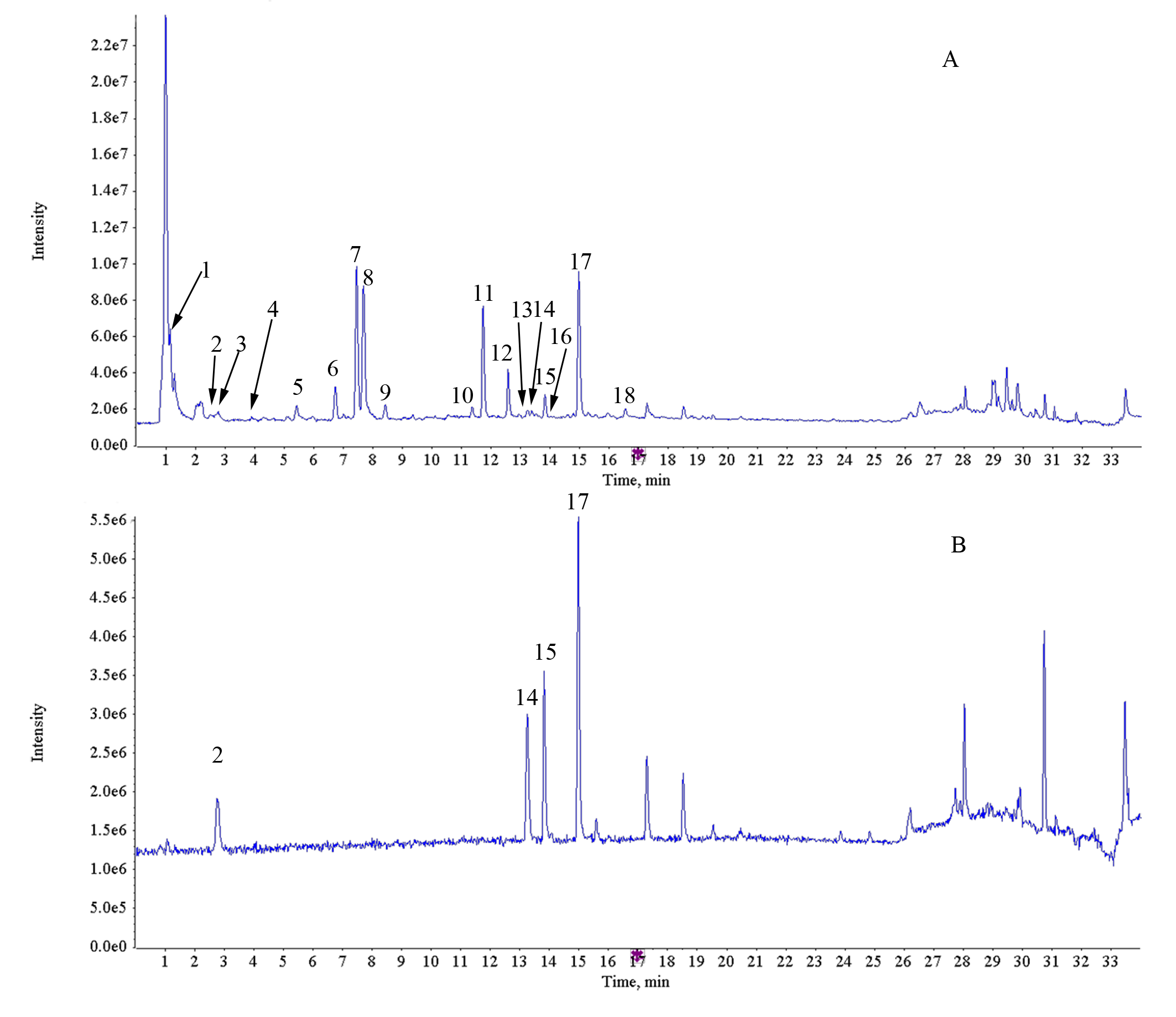


**Supplementary Figure 1.** Typical UPLC-Q-TOF chromatograms ((**A**) AEGE; (**B**) Standers).

**Supplementary Figure 2.** Chemical structures of 18 active components contained in AEGE.

**
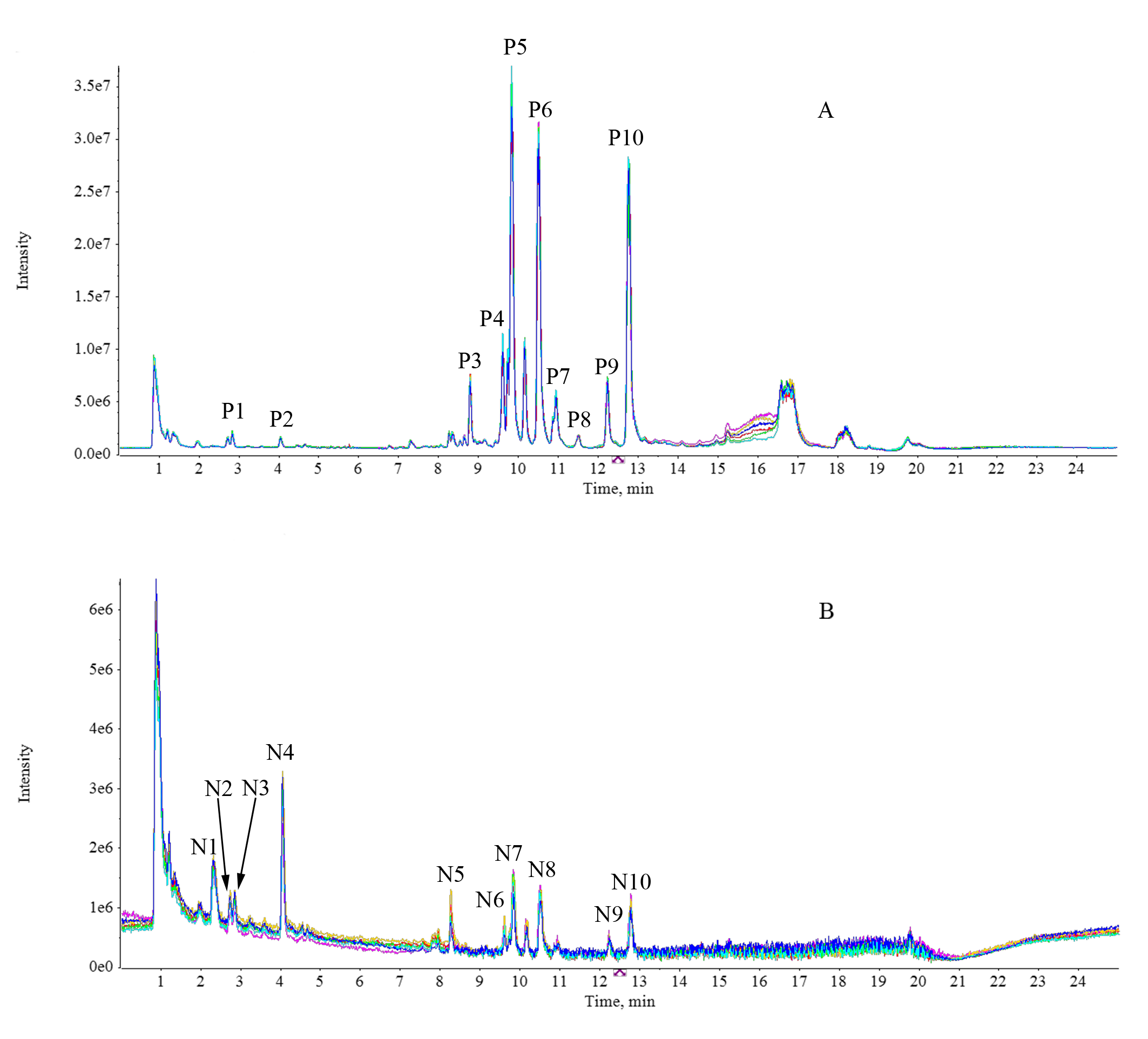
**

**Supplementary Figure 3.** TICs of six plasma QC samples ((A) positive ion mode; (B) negative ion mode.).

**
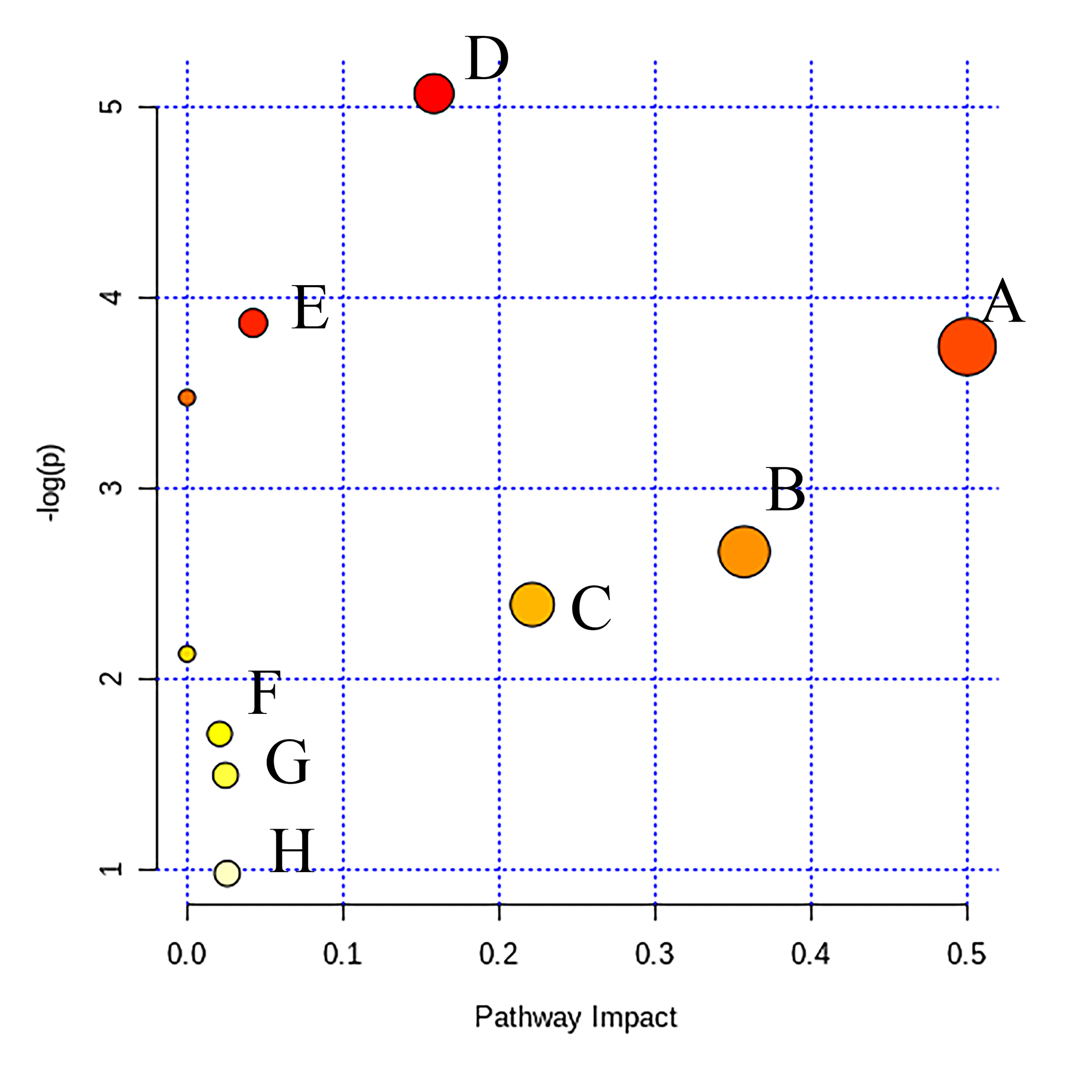
**

**Supplementary Figure 4.** Pathyway impact. **A:** Phenylalanine, tyrosine and tryptophan biosynthesis; **B:** Phenylalanine metabolism; **C:** Histidine metabolism; **D:** Sphingolipid metabolism; **E:** Pyrimidine metabolism; **F:** Cysteine and methionine metabolism; **G:** Tyrosine metabolism; **H:** Steroid hormone biosynthesis


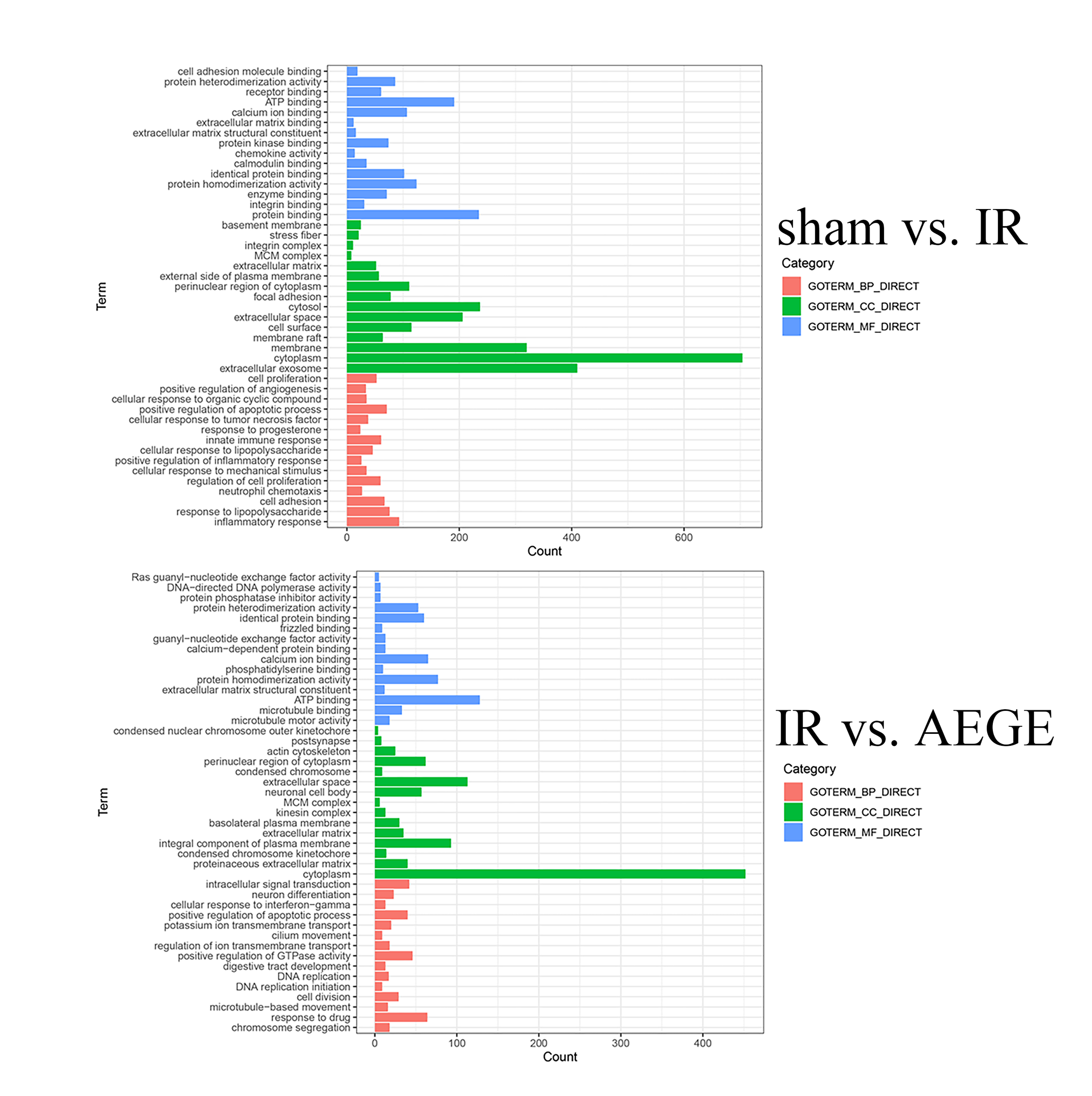


**Supplementary Figure 5.** Enriched GO terms based on DEGs. The 15 pathways enriched with the most DEGs were selected from BP, CC and MF, respectively.
