# Supplementary material for: Transcriptomic and Metabolomic Profiling Reveals the Protective Effect of Acanthopanax senticosus (Rupr. & Maxim.) Harms Combined With Gastrodia elata Blume on Cerebral Ischemia-Reperfusion Injury
Source: Front Pharmacol. 2021 Apr 16;12:619076. doi: 10.3389/fphar.2021.619076 (PMC8085551; doi:10.3389/fphar.2021.619076)
Supplement: Supplementary file 2 [file datasheet2.docx]

**Supplemental Table S1.** Identification of main components in AEGE extract based on UPLC-Q-TOF-MS

| NO | t_R_ | Name | Mass Observed | Mass Error (ppm) | Formula | Fragment ions |
| --- | --- | --- | --- | --- | --- | --- |
| 1 | 1.14 | Citric acid | 191.01973[M-H]- | 0.5 | C6H8O7 | 57.0375, 85.0312, 87.0105 |
| 2 | 2.7 | 4-Hydroxybenzyl Alcohol | 123.04515[M-H]- | 1.5 | C7H8O2 | 77.0428 |
| 3 | 2.71 | Gastrodin | 285.09798[M-H]- | -0.8 | C13H18O7 | 105.0370, 123.0448 |
| 4 | 3.91 | Protocatechuic acid | 153.01933[M-H]- | 0.9 | C7H6O4 | 81.0364, 91.0206, 109.0306 |
| 5 | 5.44 | Glucosyringic acid | 359.09837[M-H]- | 1.1 | C15H20O10 | 123.0090, 153.0556, 166.9983, 197.0458 |
| 6 | 6.73 | Ferulylquinic acid glucoside | 529.15628[M-H]- | 2.3 | C23H30O14 | 155.0349, 173.0454, 191.0556, 193.0502, 367.1038 |
| 7 | 7.45 | Parishin E | 459.11441[M-H]- | 3.4 | C19H24O13 | 87.0102, 129.0195, 173.0195, 397.1161 |
| 8 | 7.68 | Chlorogenic acid | 353.08781[M-H]- | 0.8 | C16H18O9 | 127.0403, 161.0246, 173.0455, 191.0559 |
| 9 | 8.4 | Eleutheroside B | 417.13914  [M+COOH]- | 2.2 | C17H24O9 | 151.0397, 161.0245, 179.0340, 191.0702, 209.0809 |
| 10 | 11.36 | Feruloylquinic Acid | 367.10346[M-H]- | 1.3 | C17H20O9 | 93.0357, 173.0454, 191.0560, 193.0511 |
| 11 | 11.73 | Parishin B | 727.2091[M-H]- | 6.8 | C32H40O19 | 161.0461, 369.1209, 397.1161, 423.09578, 441.1064 |
| 12 | 12.58 | Parishin C | 727.2091[M-H]- | 8.4 | C32H40O19 | 161.0460, 263.0783, 369.1218, 397.1169, 423.0966 |
| 13 | 13.24 | Quercetin | 301.03538[M-H]- | 0.4 | C15H10O7 |  |
| 14 | 13.25 | Isofraxidin | 221.04555[M-H]- | -0.5 | C11H10O5 | 135.0080, 147.0082, 163.0032,190.9983, 206.0232 |
| 15 | 13.82 | Eleutheroside E | 741.26114[M-H]- | 7.9 | C34H46O18 | 181.0490, 417.1568, 579.2159 |
| 16 | 13.92 | Hyperoside | 463.0882[M-H]- | 1.6 | C21H20O12 | 146.9409, 271.0248, 301.0384 |
| 17 | 14.97 | Parishin A | 995.30379[M-H]- | 9.8 | C45H56O25 | 161.0451, 369.1198, 423.0946, 727.2175 |
| 18 | 16.54 | Azelaic acid | 187.09758[M-H]- | 0.6 | C9H16O4 | 97.0659, 125.0966, 169.0854 |

**Supplemental Table S2.** Effects of AEGE on SOD, GSH-Px activities and IL-10, IL-1β, TNF-α, MDA contents in brain tissues of rats with CIR injuries.

| Groups | sham | IR | Nim | AEGE |
| --- | --- | --- | --- | --- |
| Dose (mg/kg) | - | - | 15 | 200 |
| SOD (U/mgprot) | 122.38±12.44 | 73.81±7.39# | 96.62±4.18* | 88.80±10.82* |
| GSH-Px (U/mgprot) | 80.26±5.13 | 43.22±7.35# | 66.23±7.43* | 60.21±6.17* |
| MDA (nmol/mgprot) | 7.61±1.52 | 11.41±1.95# | 8.93±1.38* | 8.25±1.45* |
| IL-10 (ng/L) | 615.15±66.74 | 291.83±60.92# | 440.59±106.02* | 461.45±87.67* |
| IL-1β (ng/L) | 269.47±56.15 | 454.41±51.59# | 323.50±37.63* | 359.71±55.24* |
| TNF-α (ng/L) | 1,064.59±150.56 | 1,768.82±243.06# | 1,244.35±147.08* | 1,284.94±173.83* |

‘#’ Means sham group vs. IR group (#p < 0.05); ‘*’ Means IR group vs. AEGE group (*p < 0.05).

**Supplemental Table S3**. RSD of peak areas and retention times of top 10 peaks in QC samples.

| NO. | Ion mode | M/Z | Intensity RSD(%) | Retention time RSD(%) |
| --- | --- | --- | --- | --- |
| P1 | ESI+ | 200.05 | 11.40 | 0.19 |
| P2 | ESI+ | 368.93 | 3.94 | 0.10 |
| P3 | ESI+ | 415.21 | 7.14 | 0.00 |
| P4 | ESI+ | 520.34 | 4.53 | 0.04 |
| P5 | ESI+ | 544.34 | 5.05 | 0.04 |
| P6 | ESI+ | 496.34 | 1.94 | 0.05 |
| P7 | ESI+ | 522.36 | 2.58 | 0.04 |
| P8 | ESI+ | 510.36 | 2.94 | 0.04 |
| P9 | ESI+ | 546.35 | 4.03 | 0.06 |
| P10 | ESI+ | 524.37 | 1.06 | 0.04 |
| N1 | ESI-. | 718.55 | 6.51 | 0.18 |
| N2 | ESI-. | 203.08 | 13.09 | 0.19 |
| N3 | ESI-. | 198.03 | 15.49 | 0.19 |
| N4 | ESI-. | 324.95 | 11.64 | 0.14 |
| N5 | ESI-. | 407.28 | 20.38 | 0.06 |
| N6 | ESI-. | 588.33 | 25.42 | 0.06 |
| N7 | ESI-. | 564.33 | 20.08 | 0.08 |
| N8 | ESI-. | 540.33 | 18.79 | 0.10 |
| N9 | ESI-. | 636.35 | 18.02 | 0.04 |
| N10 | ESI-. | 568.36 | 25.97 | 0.08 |

**Supplemental Table S4**. Cross-validation parameters obtained for OPLS-DA models.

| **OPLS-DA model** | **Component** | **R^2^X** | **R^2^Y** | **Q^2^** | **Permutation tests** | |
| --- | --- | --- | --- | --- | --- | --- |
|  |  |  |  |  | R^2^ | Q^2^ |
| ESI^+^ compound data | |  |  |  |  |  |
| I/R vs. Sham | 1+1 | 0.429 | 0.965 | 0.901 | 0.751 | -0.401 |
| ASE vs. I/R | 1+2 | 0.538 | 0.993 | 0.926 | 0.888 | -0.443 |
| ESI^-^ compound data | |  |  |  |  |  |
| I/R vs. Sham | 1+2 | 0.545 | 0.988 | 0.911 | 0.899 | -0.482 |
| ASE vs. I/R | 1+5 | 0.662 | 1 | 0.902 | 0.999 | -0.193 |

**Supplemental Table S5.** 116 pathways were significantly enriched by comparing sham group with IR group.

| Term | Input number | Background number | P-Value |
| --- | --- | --- | --- |
| TNF signaling pathway | 41 | 108 | 3.27E-10 |
| NF-kappa B signaling pathway | 35 | 89 | 3.03E-09 |
| Focal adhesion | 52 | 192 | 2.82E-08 |
| Pathways in cancer | 81 | 382 | 7.07E-08 |
| Proteoglycans in cancer | 52 | 201 | 9.78E-08 |
| Legionellosis | 24 | 54 | 1.5E-07 |
| Pertussis | 27 | 68 | 1.6E-07 |
| Cytokine-cytokine receptor interaction | 54 | 217 | 1.63E-07 |
| Osteoclast differentiation | 38 | 125 | 1.84E-07 |
| Malaria | 23 | 52 | 2.91E-07 |
| PI3K-Akt signaling pathway | 68 | 315 | 4.47E-07 |
| Cell cycle | 36 | 122 | 6.91E-07 |
| HTLV-I infection | 60 | 268 | 7.55E-07 |
| Complement and coagulation cascades | 27 | 77 | 1.16E-06 |
| Apoptosis | 37 | 131 | 1.17E-06 |
| Phagosome | 42 | 162 | 1.54E-06 |
| Chemokine signaling pathway | 44 | 174 | 1.54E-06 |
| AGE-RAGE signaling pathway in diabetic complications | 31 | 102 | 2.41E-06 |
| Leukocyte transendothelial migration | 33 | 114 | 2.8E-06 |
| Chagas disease (American trypanosomiasis) | 31 | 104 | 3.36E-06 |
| Tuberculosis | 41 | 164 | 4.37E-06 |
| NOD-like receptor signaling pathway | 21 | 55 | 6.06E-06 |
| Staphylococcus aureus infection | 19 | 47 | 8.67E-06 |
| Cell adhesion molecules (CAMs) | 37 | 148 | 1.25E-05 |
| Leishmaniasis | 23 | 69 | 1.38E-05 |
| Regulation of actin cytoskeleton | 46 | 206 | 1.49E-05 |
| p53 signaling pathway | 22 | 65 | 1.74E-05 |
| Salmonella infection | 25 | 81 | 1.77E-05 |
| Platelet activation | 31 | 115 | 1.82E-05 |
| Hematopoietic cell lineage | 25 | 82 | 2.11E-05 |
| Small cell lung cancer | 25 | 83 | 2.49E-05 |
| Measles | 32 | 125 | 3.18E-05 |
| Amoebiasis | 27 | 96 | 3.32E-05 |
| ECM-receptor interaction | 23 | 75 | 4.11E-05 |
| Epstein-Barr virus infection | 44 | 203 | 4.18E-05 |
| Oxytocin signaling pathway | 36 | 152 | 4.32E-05 |
| Natural killer cell mediated cytotoxicity | 26 | 93 | 5.01E-05 |
| DNA replication | 15 | 36 | 5.73E-05 |
| HIF-1 signaling pathway | 27 | 102 | 7.95E-05 |
| MAPK signaling pathway | 50 | 251 | 8.51E-05 |
| Viral myocarditis | 21 | 70 | 0.000113 |
| Rheumatoid arthritis | 23 | 85 | 0.000199 |
| Transcriptional misregulation in cancer | 35 | 161 | 0.000231 |
| Toll-like receptor signaling pathway | 24 | 93 | 0.000267 |
| Viral carcinogenesis | 40 | 200 | 0.000386 |
| Herpes simplex infection | 38 | 187 | 0.000408 |
| Influenza A | 34 | 161 | 0.000444 |
| cAMP signaling pathway | 38 | 189 | 0.000488 |
| Rap1 signaling pathway | 40 | 203 | 0.000499 |
| Hepatitis B | 30 | 136 | 0.000507 |
| Toxoplasmosis | 27 | 118 | 0.000585 |
| Calcium signaling pathway | 35 | 171 | 0.000604 |
| Fc gamma R-mediated phagocytosis | 21 | 83 | 0.000779 |
| EGFR tyrosine kinase inhibitor resistance | 20 | 80 | 0.001162 |
| Cytosolic DNA-sensing pathway | 15 | 52 | 0.001434 |
| Jak-STAT signaling pathway | 30 | 147 | 0.001483 |
| B cell receptor signaling pathway | 18 | 71 | 0.001757 |
| Primary immunodeficiency | 12 | 37 | 0.001863 |
| Dilated cardiomyopathy | 20 | 84 | 0.001913 |
| Apoptosis - multiple species | 11 | 32 | 0.001957 |
| Bacterial invasion of epithelial cells | 19 | 78 | 0.001966 |
| Hypertrophic cardiomyopathy (HCM) | 19 | 80 | 0.00251 |
| Central carbon metabolism in cancer | 16 | 62 | 0.002654 |
| Phospholipase D signaling pathway | 28 | 141 | 0.002934 |
| Pancreatic cancer | 16 | 63 | 0.003037 |
| Ras signaling pathway | 39 | 220 | 0.003124 |
| Adipocytokine signaling pathway | 17 | 72 | 0.004334 |
| Gastric acid secretion | 16 | 66 | 0.004472 |
| Platinum drug resistance | 18 | 79 | 0.004668 |
| Metabolic pathways | 159 | 1202 | 0.00467 |
| Melanoma | 16 | 68 | 0.005705 |
| African trypanosomiasis | 11 | 38 | 0.005816 |
| Axon guidance | 31 | 172 | 0.006356 |
| Purine metabolism | 31 | 172 | 0.006356 |
| Salivary secretion | 16 | 70 | 0.0072 |
| Cysteine and methionine metabolism | 12 | 46 | 0.008053 |
| Antigen processing and presentation | 17 | 78 | 0.008485 |
| Glioma | 14 | 60 | 0.009931 |
| Estrogen signaling pathway | 19 | 93 | 0.00996 |
| Pyrimidine metabolism | 20 | 100 | 0.010111 |
| Cholinergic synapse | 21 | 107 | 0.010194 |
| Graft-versus-host disease | 12 | 48 | 0.010578 |
| Choline metabolism in cancer | 19 | 96 | 0.013086 |
| Arrhythmogenic right ventricular cardiomyopathy (ARVC) | 15 | 69 | 0.01316 |
| Arginine and proline metabolism | 12 | 50 | 0.013674 |
| Intestinal immune network for IgA production | 11 | 44 | 0.014059 |
| Circadian entrainment | 18 | 90 | 0.01418 |
| Adherens junction | 15 | 70 | 0.014604 |
| VEGF signaling pathway | 13 | 58 | 0.016527 |
| Endocrine resistance | 18 | 93 | 0.018455 |
| One carbon pool by folate | 6 | 17 | 0.018659 |
| Base excision repair | 9 | 34 | 0.018952 |
| ErbB signaling pathway | 17 | 87 | 0.020092 |
| Hepatitis C | 22 | 124 | 0.02212 |
| RIG-I-like receptor signaling pathway | 13 | 61 | 0.022803 |
| FoxO signaling pathway | 22 | 125 | 0.023737 |
| Insulin secretion | 16 | 82 | 0.023871 |
| Colorectal cancer | 13 | 62 | 0.025244 |
| Type I diabetes mellitus | 12 | 56 | 0.027135 |
| Renin secretion | 13 | 63 | 0.027872 |
| Bladder cancer | 9 | 37 | 0.028605 |
| Allograft rejection | 11 | 50 | 0.029029 |
| T cell receptor signaling pathway | 19 | 106 | 0.029335 |
| Oocyte meiosis | 19 | 106 | 0.029335 |
| Insulin resistance | 19 | 107 | 0.031551 |
| Inflammatory mediator regulation of TRP channels | 19 | 107 | 0.031551 |
| Biosynthesis of amino acids | 15 | 80 | 0.036605 |
| MicroRNAs in cancer | 34 | 225 | 0.037284 |
| Glutathione metabolism | 11 | 53 | 0.039751 |
| Chronic myeloid leukemia | 14 | 74 | 0.040164 |
| Amphetamine addiction | 12 | 61 | 0.044179 |
| Insulin signaling pathway | 21 | 127 | 0.044256 |
| Sphingolipid signaling pathway | 20 | 120 | 0.045889 |
| Ether lipid metabolism | 9 | 41 | 0.046207 |
| Inflammatory bowel disease (IBD) | 12 | 62 | 0.048317 |
| Alanine, aspartate and glutamate metabolism | 8 | 35 | 0.04932 |

**Supplemental Table S6.** 28 pathways were significantly enriched by comparing AEGE group with IR group.

| Term | Input number | Background number | P-Value |
| --- | --- | --- | --- |
| Cell cycle | 24 | 122 | 1.01053E-05 |
| DNA replication | 12 | 36 | 2.54277E-05 |
| Cell adhesion molecules (CAMs) | 24 | 148 | 0.000152106 |
| HTLV-I infection | 35 | 268 | 0.00027231 |
| Hippo signaling pathway | 21 | 146 | 0.001506251 |
| Wnt signaling pathway | 20 | 139 | 0.001919723 |
| AGE-RAGE signaling pathway in diabetic complications | 16 | 102 | 0.002443724 |
| Leukocyte transendothelial migration | 17 | 114 | 0.002917308 |
| Proteoglycans in cancer | 25 | 201 | 0.003431219 |
| Viral myocarditis | 12 | 70 | 0.004368343 |
| Basal cell carcinoma | 10 | 52 | 0.004425245 |
| Staphylococcus aureus infection | 9 | 47 | 0.006940396 |
| Neuroactive ligand-receptor interaction | 30 | 273 | 0.007350769 |
| Axon guidance | 21 | 172 | 0.008313142 |
| Purine metabolism | 21 | 172 | 0.008313142 |
| Pyrimidine metabolism | 14 | 100 | 0.010533251 |
| Pathways in cancer | 38 | 382 | 0.011920616 |
| Hedgehog signaling pathway | 8 | 43 | 0.01228803 |
| Regulation of actin cytoskeleton | 23 | 206 | 0.014834606 |
| Fanconi anemia pathway | 8 | 47 | 0.01893704 |
| Tight junction | 16 | 134 | 0.023074799 |
| Signaling pathways regulating pluripotency of stem cells | 16 | 137 | 0.027176002 |
| Fc gamma R-mediated phagocytosis | 11 | 83 | 0.030013267 |
| Cytosolic DNA-sensing pathway | 8 | 52 | 0.030431343 |
| Malaria | 8 | 52 | 0.030431343 |
| Oxytocin signaling pathway | 17 | 152 | 0.03236723 |
| Base excision repair | 6 | 34 | 0.034713206 |
| Calcium signaling pathway | 18 | 171 | 0.044837823 |
